# Understanding Placebo and Nocebo Responses Based on a Randomized Sham-Controlled Study on Acupuncture in Integrative Cancer Care

**DOI:** 10.1177/15347354241300068

**Published:** 2024-11-16

**Authors:** Anna Efverman

**Affiliations:** 1 University of Gävle, Gävle, Sweden

**Keywords:** acupuncture, cancer, contextual effects, traditional, complementary, and integrative medicine, expectations, nursing, oncology, psycho-oncology

## Abstract

**Objective:** Since placebo and nocebo-responses during acupuncture therapy are rarely studied in clinical contexts, the objective was to investigate placebo and nocebo-responses in a clinical context through comparing positive and negative side-effects between genuine and sham acupuncture, and to identify factors modifying placebo and nocebo-responses. **Methods:** Patients reported positive side-effects (non-intended positive effects) or negative side-effects (non-intended negative effects) of genuine (penetrating; n = 109) or sham (telescopic non-penetrating; n = 106) acupuncture during 5 weeks of chemoradiation for cancer. **Results:** The genuine and the sham acupuncture group reported rather similar frequencies of positive *(P*-values .223-.800) or negative (*P*-values .072-1.0) side-effects: relaxation 59%/57% of the patients, improved mood 46%/38%, improved sleep 38%/38%, pain-reduction 36%/28%, tiredness 42%/42%, feeling cold 37%/31%, sweating 23%/21%, and dizziness 20%/12%. Positive side-effects occurred in 79% of patients who highly believed acupuncture to be effective, compared to in 0% of patients who did not believe. Other factors associated with placebo-response were female sex (*P* = .042), anxious mood (*P* = .007), depressed mood (*P* = .018), and blinding-success (*P* = .033). Factors associated with nocebo response were female sex (*P* = .049), younger age (*P* = .010), and needle-induced pain (*P* = .014). Sham-treated patients experiencing positive side-effects experienced better quality of life at the end of the treatment period (mean, m 64 on a scale 0-100 millimetres, Standard Deviation, SD, ±26.1 mm) than those who did not (m 48 ± 25.5 mm), *P* = .048 (adjusted for other characteristics). **Conclusions:** Clinically significant levels of placebo and nocebo effects commonly occurred during acupuncture therapy in integrative cancer care and this paper identified factors modifying these responses. This implicates that acupuncture-delivering therapists striving to maximize placebo-responses and minimize nocebo-responses may considering strengthen the patient’s treatment expectations, and offer a pleasant, pain-free, acupuncture treatment.

## Introduction

Cancer care professionals in integrative oncology^1,2^ should be aware of the potential health effects of factors surrounding the given treatment^3-6^ to be able to maximize benefits of therapies and minimize negative side-effects.^2^ However, clinical research has yet mainly focused on the specific effects of treatments while placebo and especially nocebo-responses are more rarely studied.^3,7-10^ Implications of placebo and nocebo responses for clinical practice are based mostly on experimental studies and more studies from clinical practice are welcomed.^3,6,10,11^

In an overview of systematic reviews on complementary and alternative medicine in patients with cancer, acupuncture was the most studied therapy, covering more than a fifth of 104 reviews.^12^ Within the 2 past decades, research on acupuncture therapy in cancer care has rapidly increased^13^ and acupuncture therapy is on the top-3 list of complementary and alternative medicine therapies integrated at oncology care clinics worldwide.^14,15^ Integrative oncology^1,2^ strives to integrate conventional medical therapies and complementary and alternative therapies that together bring the greatest summed health benefits, in terms of maximized treatment effects and positive non-intended side-effects and minimized negative side-effects.^2^

Both conventional medicine and integrative medicine therapies can be divided into having specific genuine treatment components and non-specific components including the components surrounding the treatment, often referred to as the placebo components.^3,6^ Such components may include the interaction between the patient and the therapist,^5,7^ the environment, and treatment expectations.^3,6^ Conditioning of positive treatment responses through learning from previous experiences,^16^ and treatment expectation of positive treatment outcomes^5,17^ or of chance to recover^18^ are suggested to be dominating components contributing to the placebo response.^3,6^ Nowadays, placebo responses are defined as positive treatment outcomes that cannot be attributed to active treatment components but are elicited by positive expectations or the psychosocial context in which treatment takes place.^3,6^

On the opposite, the nocebo response involves unwanted, negative consequences of non-specific components of treatments, driven by learning from negative experiences resulting in negative treatment expectations or attributed to the psychosocial context of the treatment.^8^ The effects of previous experiences and treatment expectations per se have previously mostly been studied in relation to the treated outcome, in other words, the *intended* effect.^3,6^ However, *other* effects than the intended may also occur during treatments. These effects may be presented as negative side-effects, depending on if the non-intended effects seem harmful or beneficial. A positive side-effect may be defined as a positive, that is, beneficial, effect on other outcomes than the outcome intended to treat. A negative side-effect may be defined as a negative, that is, worsening, or harmful, effect on other outcomes than the outcome intended to treat.^19^ When these positive and negative side-effects occur during a sham therapy that does not include any specific mechanisms for delivering such effects, they may be addressed to as placebo and nocebo responses.^3,6-8^

A relevant clinical context for studying placebo and nocebo responses may be acupuncture therapy, since it is previously known that acupuncture therapy procedures include known nonspecific treatment components^20-23^ and it is possible to successfully blind study participants.^24^ Of 927 identified acupuncture trials on acupuncture therapy in cancer care, very few explored placebo and nocebo effects of symptom-managing acupuncture therapy in cancer care.^13^ Acupuncture was associated with greater placebo responses and smaller nocebo responses than were pills when treating breast cancer diagnosed women’s hot flashes.^25^ A previous paper reported the intended primary outcomes from a randomized sham-controlled trial (RCT). Genuine acupuncture did not reduce nausea and vomiting more than non-penetrating sham acupuncture in successfully blinded patients.^26^ This finding, in convergence with reports from patients who propose non-specific treatment components to contribute to their experience of acupuncture during and after cancer therapies,^27,28^ raise interest in reporting secondary outcomes of the RCT^26^ by studying placebo and nocebo responses during acupuncture therapy in integrative oncology care.^1,2^ The objective of this paper was to investigate placebo and nocebo responses in a clinical cancer care context through comparing positive and negative side effects between genuine and non-penetrating sham acupuncture treated patients, and to identify factors associated with placebo and nocebo responses of sham acupuncture.

## Methods

### Design and Setting

This paper reports secondary outcomes from the original prospective RCT mentioned above.^26^ The study was registered (ClinicalTrials.gov), adhered to the declaration of Helsinki, and the regional ethics committee of Linköping approved the study (approval number 02-420, M167-04, dates 2002-11-05 and 2004-12-14). The original RCT covered 215 consecutively included patients with cancer undergoing radiotherapy at the oncology clinics of 2 Swedish university hospitals. The radiotherapy was given with or without concomitant chemotherapy. By use of computerized random table, the patients were randomized between genuine or sham acupuncture to treat—as previously reported—the RCT’s primary outcome, radiotherapy-induced nausea.^26^

### Study Criteria

This paper covered all patients included in the original RCT.^26^ Inclusion criteria were an age of at least 18 years, a gynecologic, anal, rectal, colon, stomach, pancreatic or testicular cancer, and radiotherapy to pelvic or abdominal fields of at least 800 cm^3^ volume and 25 Gy dose. Only patients with ability to give informed consent were included, meaning that patients with very poor physical or mental condition were excluded, for example patients severely sedated and confused due to their cancer illness or mental illness. Other exclusion criteria were persistent emesis or consuming antiemetics already before start of radiotherapy (within 24 hours), acupuncture therapy during the past year regardless of indication, or had received antiemetic acupuncture therapy any time.

Of 522 screened patients, 169 did not meet study criteria, 138 did not want to participate, while 215 were included. All patients received written and oral information and gave informed consent.^26^ The letter informed that the effect of either needling type was unknown, and that: “Potential side-effects of the acupuncture treatments will be registered during every treatment session. The side-effects may be for example soreness around the needling point, hematoma, tiredness or, more unusual, dizziness.”

### Genuine and Sham Acupuncture Treatment

Therapists delivered genuine and sham acupuncture therapy for 30 minutes 2 to 3 times a week during the median 5 weeks of radiotherapy. Western medical manual genuine acupuncture^26^ was given using “deqi”-inducing sharp needles placed in pericardium six, PC6, bilaterally. Sham acupuncture was given using Park’s non-penetrating credible telescopic sham device.^29^ Except for placing and manipulating the needle (three times a treatment), the sham needle induced no pressure against the skin at all. This implies that the blunt needles produced no “acupressure.”^30^ For detailed information regarding the therapy and the therapists, please see the original report.^26^

### Data Collection Method for Assessment of Demographics, Treatment-Expectations, and Other Characteristics of the Patients

The study coordinating nurse collected medical record data (Table 1). At baseline, that is, the day before the first genuine or sham acupuncture treatment, the patients in writing in privacy delivered sociodemographic information. They reported if they previously had received acupuncture, and graded their level of anxious mood (“Have you within the past month experienced anxious mood?”: “No,” “Yes, a little,” “Yes, moderately,” “Yes, much”), and depressed mood (“Have you within the past month experienced depressed mood?”: “No,” “Yes, a little,” “Yes, moderately,” “Yes, much”).^31^ The patients graded quality of life (“How did you perceive your quality of life?”) using verbal categories (“High,” “Moderate,” “Low” and “Very low; non-existent quality of life”), and using a Visual Analog Scale (VAS), ranged 0 (worst imaginable; no-existing quality of life) to 100 (best imaginable) millimeters (mm).^32^ The patients stated “totally disagree,” “partly disagree,” partly agree” or “totally agree” regarding the single statement “I try to be optimistic,” derived from the Mental Adjustment to Cancer scale.^33^ Immediately after the first, the middle (the 6th) and the last (the 12th) treatment, the treating therapist asked: “Do you think that the treatment that you just received is effective to prevent and reduce nausea?” (“No, I do not think the treatment is effective” or “Yes, I believe a little”/“moderately”/“much that the treatment is effective”).^34^ After the last treatment, the questionnaire again asked the patients to grade their quality of life.^32^

**Table 1.** Demographics of the Patients in the Genuine and the Acupuncture Group.

Variable	Totals n = 215	Genuine acupuncture n = 109	Sham acupuncture n = 106
Sex, n (%)			
Male	35 (16)	20 (18)	15 (14)
Female	180 (84)	89 (82)	91 (86)
Age in years m ± SD, range	63.7 ± 13.8, 22-91	64.3 ± 13.8, 22-91	63.0 ± 13.9, 25-89
Cancer type, n (%)			
Gynecological-	147 (68)	72 (66)	75 (71)
Colon-, rectal- or anal-	60 (28)	31 (28)	29 (27)
Testicular-	2 (1)	2 (2)	0 (0)
Pancreas- or ventricular-	6 (3)	4 (4)	2 (2)
Clinical status, n (%)			
Treated as out-patient	175 (81)	91 (83)	84 (79)
Treated as in-patient at least 1 wk	40 (19)	18 (17)	22 (21)
Irradiated site, n (%)			
Abdomen	13 (6)	6 (6)	7 (7)
Pelvis	202 (94)	103 (94)	99 (93)
Radiated dose (Gray), m ± SD Range	49.1 ± 10.6, 2.00-71.2	47.9 ± 10.7, 2.00-70.0	50.3 ± 10.3, 6.0-71.2
Radiated volume (cm^3^), m ± SD	1424 ± 608	1414 ± 616	1434 ± 602
Length of radiotherapy period in days, m ± SD	36 ± 10	36 ± 10	36 ± 9
Concomitant chemotherapy during radiotherapy, n (%)	n = 199	n = 100	n = 99
Yes/No	57 (29)/ 142 (71)	28 (28)/ 72 (72)	29 (29)/ 70 (71)
Have received acupuncture before,^a^ n (%)	n = 210	n = 108	n = 102
Yes/No	72 (34)/138 (66)	36 (33)/72 (67)	36 (35)/66 (65)
Marital status, n (%)	n = 192	n = 97	n = 95
Married or living togethe	120 (62)	60 (62)	60 (63)
Living alone, have a partner	12 (6)	7 (7)	5 (5)
Living alone, single	60 (31)	30 (31)	30 (32)
Employment status, n (%)	n = 210	n = 106	n = 104
Employed	76 (36)	35 (33)	41 (38)
Student	2 (1)	1 (1)	1 (1)
Housewife/male homemaker	4 (2)	1 (1)	3 (3)
Retired or sickness pension	128 (61)	69 (65)	59 (57)
Medication for co-morbidity, n (%)	n = 199	n = 99	n = 100
Yes	168 (84)	80 (81)	88 (88)
No	31 (16)	19 (19)	12 (12)

Numbers (n) of patients answering the questions are presented.

Abbreviations: M, mean; SD, standard deviation.

a The indications for the past acupuncture experiences in the 72 patients were: pain (n = 57; 79%), abstinence (n = 4; 6%), anxiety or depression (n = 3; 4%), tinnitus (n = 1; 1%), weight reduction (n = 1; 1%), hot flushes (n = 1; 1%), and five did not specify indication.

### Data Collection Methods for Registration of Side-Effects

Using 2 data collection methods for asking the patients to register side-effects give opportunities to investigate data collection method as a potential factor for modifying occurrence of placebo and nocebo effects.

Registering of side-effects occurring *during* treatments: At the end of every genuine and sham acupuncture treatment, the therapists asked and inspected the patients regarding potential occurrence (“Yes” or “No”) of the exemplified negative side-effects^35^ tiredness, dizziness, fainting, and registered them in a treatment protocol. The therapist asked the patients to describe, using their own words, if they had noticed any other negative side-effects, or had experienced any positive sensations during the treatment.

Registering of side-effects occurring *close to* treatments: Every seventh day during the treatment period, the patients answered 5 questions using *structured answering alternatives*^35^: “Have you within or close to the acupuncture sessions experienced any negative side-effects regarding soreness around the needling points/sweating/tiredness/dizziness/feeling cold” (“Yes” or “No”). The patients also provided answers to 2 questions, using *their own words*: “If you within or close to the acupuncture sessions experienced any negative side-effects, what side-effects?,” “If you within or close to the acupuncture sessions experienced any negative side-effects, what side-effects?.” The patients answered the same questions^34^ regarding positive side-effects.

### Statistical Analyses

The evaluator calculated the proportion of patients experiencing the variety of side-effects exemplified using the structured answering alternatives, and described by the patients using their own words, and compared the proportions using chi-squared test. Chi- squared tests were used to compare the genuine and sham group regarding occurrence of the different exemplified side-effects at least once within the treatment period. Comparisons were presented as Relative Risk (RR) for side-effects, with 95% Confidence Intervals (CI). Placebo responders were defined as the proportion experiencing positive side-effects after receiving sham acupuncture, and nocebo responders as the proportion in the sham group experiencing negative side effects, in line with how other researchers categorized the responses.^36^ Based on previous literature on potential factors modifying placebo and nocebo responses,^3,6-10^ variables potentially explaining the variation in placebo and nocebo responses were selected: sex, age, previous acupuncture experience, anxious mood, depressed mood, quality of life, treatment expectancy, optimism, blinding statement, needle-induced pain, and the acupuncture-treating therapist. Then the patients with different characteristics were compared, regarding occurrence of: (1) Negative side-effects of sham acupuncture (“nocebo responses”); (2) Positive effects of sham acupuncture (“placebo responses”); (3) Negative treatment effects of genuine acupuncture; and (4) Positive treatment effects of genuine acupuncture. For these comparisons, the evaluator used Chi-squared test regarding nominal variables (Fisher’s exact test if n < 5 within at least one category), Mann Whitney *U*-test regarding ordinal variables, and Student’s t-test regarding continuous normally distributed data, and regarding the variable VAS, since previous studies feasibly analyzed VAS as being a continuous variable.^37^ When comparing sham-acupuncture treated patients experiencing and not experiencing positive side-effects regarding perceived quality of life, a linear regression model analyzed whether the difference remained after controlling for other independent variables that reasonable may affect quality of life: sex, age, cancer type, and the other variables shown in Table 1 (previous acupuncture experiences excluded). The analyses were performed in Statistical Package for the Social Sciences (IBM SPSS Statistics for Windows), version 23 (IBM Corp. Armonk, NY, USA). A 5% significance level was set, with *P* < .05 as a statistically significant difference.

## Results

### The Patients

Of the 215 patients starting acupuncture, 109 received genuine acupuncture (97 patients completed), and 106 received sham acupuncture (100 patients completed). The 215 patients received a total of 2426 treatments: 1154 genuine and 1248 sham acupuncture treatments (median 11 treatments per patient). Approximately one-third of the patients had previously received acupuncture (Table 1). Table 1 presents other characteristics of the participants. The patients were, as reported previously,^26^ successfully blinded (92% of 95 answering patients in the genuine acupuncture group and 81% of 95 answering patients in the sham acupuncture group believed that they had been treated with penetrating needles).

### Negative Side-Effects in Patients Receiving Genuine or Sham Acupuncture

*During treatments*, there were no difference between the genuine and the sham acupuncture groups regarding occurrence of negative side-effects. There was one exception; numbness occurred more often during genuine acupuncture (Table 2).

**Table 2.** Side-Effects During and Close to Genuine and Sham Acupuncture.

Variable	Totally	Genuine acupuncture	Sham acupuncture	*P*-value
Negative side-effects *during* treatment, n (%) of treatments	n = 2426 treatments	n = 1178 treatments	n = 1248 treatments	
Tiredness	55 (2.3)	35 (3.0)	20 (1.6)	.376
Dizziness	20 (0.8)	14 (1.2)	6 (0.5)	.287
Fainting	0	0	0	NA
Extremely unpleasant	11 (0.5)	11 (0.9)	0	.185
Irradiating sensations	11 (0.5)	9 (0.8)	2 (0.2)	
Numbness	6 (0.2)	6 (0.5)	0	.035*
Nausea	4 (0.2)	2 (0.2)	2 (0.2)	NA
Sweating	2 (0.1)	0	2 (0.2)	NA
Feeling cold	3 (0.1)	2 (0.2)	1 (0.1)	NA
Soreness in the wrist or hand	3 (0.1)	3 (0.3)	0	NA
Cold hands	1 (0.04)	1 (0.1)	0	NA
Negative side-effects *close to* the treatments,^a^ mentioned by the patients using their own words, n of patients	n = 215	n = 109	n = 106	
Unpleasant	3	3	0	NA
Irradiating sensations	1	1	0	NA
Spasm in the hand	1	1	0	NA
Needling induced nausea	1	1	0	NA
Headache	1	0	1	NA
Positive side-effects *during* treatment, mentioned by the patients using their own words, n (%) of treatments	n = 2426 treatments	n = 1178 treatments	n = 1248 treatments	
Relaxation	27 (1.1)	12 (1)	15 (1.2)	.696
Increased psychological energy	5 (0.2)	5 (0.4)	0	.232
Pain-reduction	2 (0.1)	1 (0.1)	1 (0.1)	NA
Reduced anxiety	2 (0.1)	1 (0.1)	1 (0.1)	NA
Feeling warm	1 (0.04)	1 (0.1)	0	NA
Reduced diarrhea	1 (0.04)	1 (0.1)	0	NA
Reduced hot flushes	1 (0.04)	1 (0.1)	0	NA
Increased well-being	1 (0.04)	1 (0.1)	0	NA
Positive side-effects *close to* treatments^a ^mentioned by the patients using their own words, n of patients	n = 215	n = 109	n = 106	
Increased wellbeing	3	1	2	NA
Increased psychological energy	3	1	2	NA
Relief of numbness in the legs	3	2	1	NA
Relief of anxiety	2	1	1	NA
Time for resting/taking a pause	2	1	1	
Warm hands	2	1	1	
Pleasant drowsiness	1	0	1	NA
Improved mobility in the hand	1	0	1	NA
Relief of metallic taste in the month	1	1	0	NA
Improved hearing	1	1	0	NA
Improved visual perception	1	1	0	NA

Numbers and proportions (%) of treatments and n of patients are presented.

NA = hypothesis testing was not performed, due to low number of reports. Another comments were: Valuable conversations with the therapist; n = 4 genuine and n = 4 sham acupuncture treated patients, and Long duration of sitting/laying during needling; n = 5 genuine acupuncture treated patients.

a Side-effects other than those exemplified in the questionnaire.

* Statistically significant difference between the genuine and sham acupuncture group.

**Figure 1.**
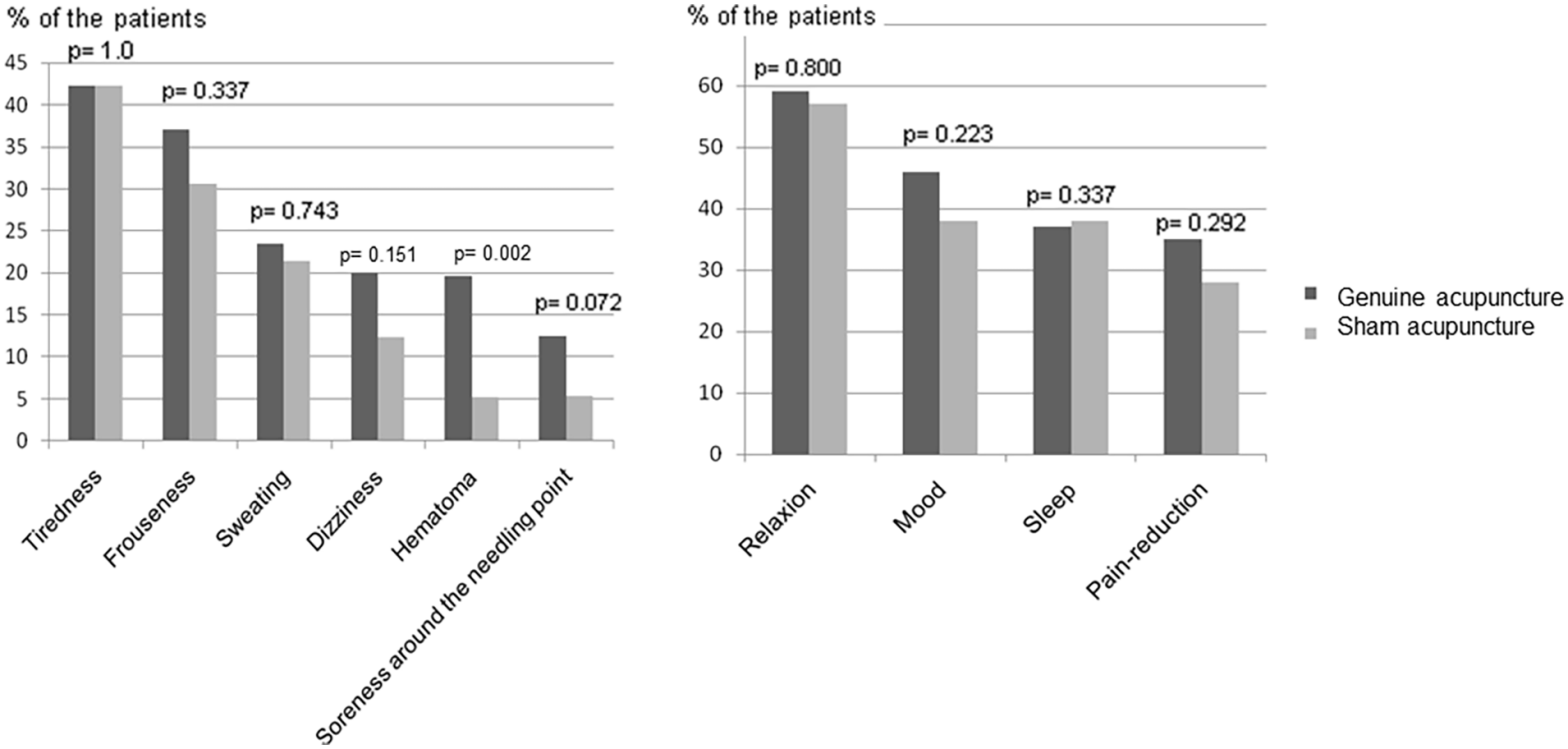
Negative and positive side-effects close to the treatments occurring at least once during the treatment period in the genuine and sham acupuncture group.

*Close to treatments*, there were no statistically significant differences between the genuine and the sham acupuncture group regarding occurrence of negative side-effects, except hematoma; bleeding cannot occur during non-penetrating sham acupuncture (Figure 1). Tiredness was the most frequently experienced negative side-effect in both groups, occurring close to at least one treatment session in 42% of both the genuine and sham acupuncture treated patients. Hematoma around the needling point, because of a small bleeding from penetrating needles, is not possible to occur during non-penetrating sham acupuncture. However, 5% of the sham treated patients suggested that they received hematoma (Figure 1). Nor did any differences occur between the groups when observing separate weeks of the study. The patients mentioned other negative side-effects using their own words (Table 2), which occurred less frequently than those exemplified in the questionnaire covering structured answering alternatives (Figure 1).

### Positive Side-Effects in Patients Receiving Genuine or Sham Acupuncture

There were no statistically significant differences between the genuine and the sham acupuncture group regarding occurrence of any of the positive side-effects occurring either *during* (Table 2) or *close to* treatments within the entire treatment period (Figure 1). The most common positive side-effect reported during treatment and close to treatments was relaxation (Table 2 and Figure 1).

### Experienced Side-Effects Depending on the Type of Data Collection Method

The proportions who reported side-effects measured using structured answering alternatives differed compared to the proportions who reported side-effects measured using the patients’ own words. Based on the patients’ *own words*, any kind of positive side-effects occurred at least once in 12 (11%) of the 109 genuine acupuncture treated patients and in 8 (8%) of the 106 sham treated patients (*P* = .482). Based on the *structured answering alternatives* covering examples of potential side-effects, any kind of positive side-effects occurred in 64 (59%) of the genuine and in 65 (61%) of the sham acupuncture treated patients (*P* = .876). Thus, the proportion of patients who reported positive side-effects in the genuine acupuncture group was 5.3 (RR) times higher (CI 3.06-9.30) when using structured examples than assessed using the patients’ own worlds. The corresponding figures for the sham acupuncture group was 8.13 (RR) times higher (CI 4.10-16.09).

### Treatment Expectations as a Factor Modifying Placebo Responses

**Figure 2.**
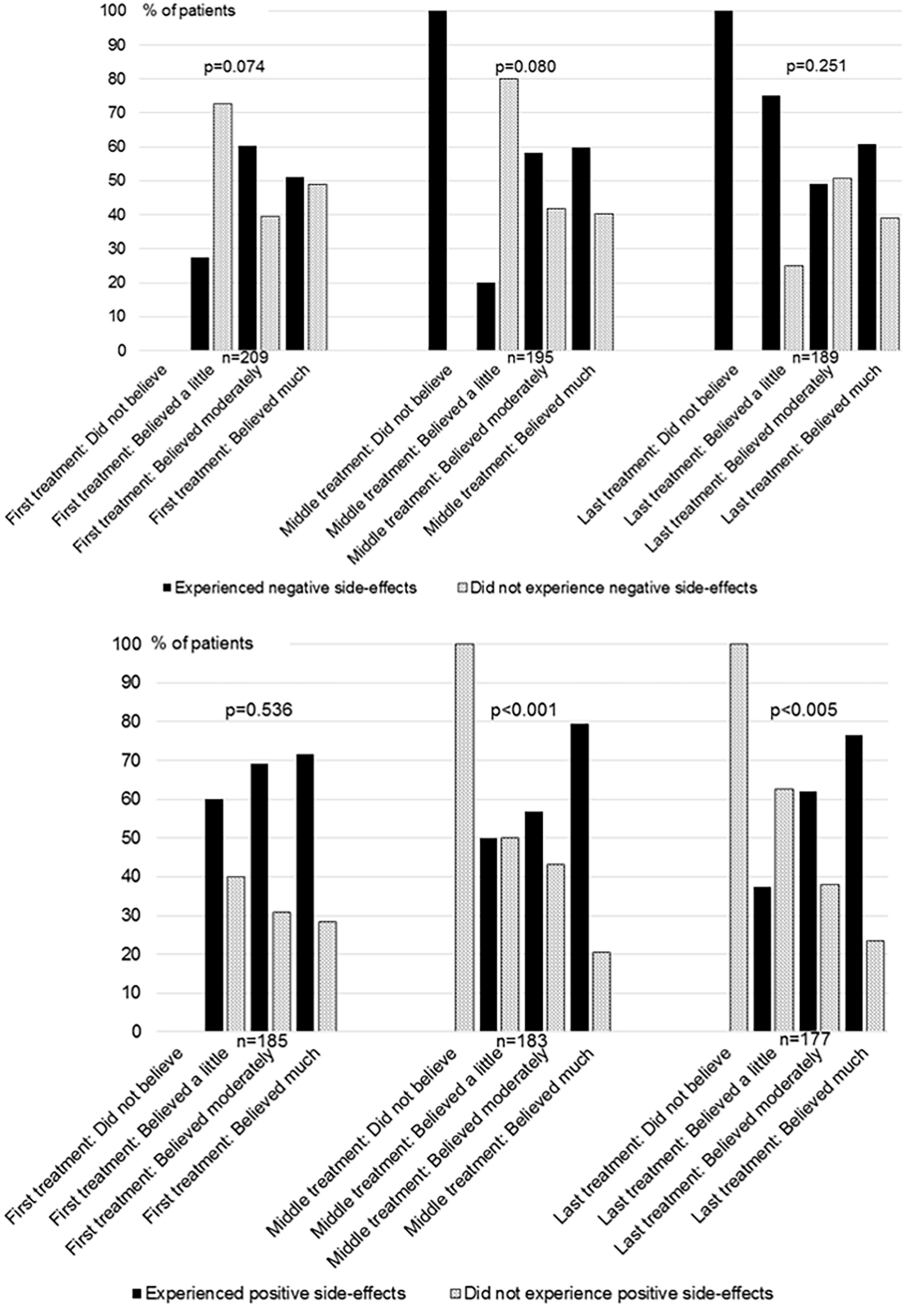
Occurrence of negative and positive side-effects close to the treatments in patients expressing different levels of treatment-expectations, that is, belief in the efficacy of the treatment. The numbers of patients delivering data regarding both belief and side-effects are presented.

At baseline, all patients to some degree believed that acupuncture would be effective, no patients stated that they did not believe acupuncture to be effective. Positive side-effects occurred in 79% of patients who highly believed acupuncture to be effective in the middle of the acupuncture treatment period, compared to 0% of the patient who did not believe (*P* = .001). Positive side-effects occurred in 76% of patients who highly believed acupuncture to be effective at the last treatment, compared to 0% of the patient who did not believe (*P* = .005; Figure 2).

### Factors Modifying Placebo Responses During Sham Acupuncture

More women reported positive (*P* = .020) side-effects of sham acupuncture than the men did. More of the patients who experienced anxious mood (*P* = .014), or depressed mood (*P* = .018), reported positive side-effects than patients who did not experience anxious or depressed mood. More of the patients who believed that they had received penetrating acupuncture reported positive side-effects (*P* = .033) than others, irrespective of if they received penetrating genuine acupuncture or not (Table 3).

**Table 3.** Occurrence of Negative and Positive Side-Effects During Genuine or Sham Acupuncture in Patients With Different Baseline Characteristics.

	Genuine acupuncture n = 109	Sham acupuncture n = 106	Genuine acupuncture n = 109	Sham acupuncture n = 106
Variable	Yes, negative side-effects occurred, n = 71	Yes, negative side-effects occurred, n = 46	Yes, positive side-effects occurred, n = 64	Yes, positive side-effects occurred, n = 65
*Individual factors*				
Sex, n (%)	*P* = .611	*P* = .049*	*P* = .270	*P* = .020*
Man	12 (60)	4 (27)	10 (56)	5 (39)
Woman	59 (66)	42 (47)	54 (70)	60 (74)
Age in years^a^	*P* = .200	*P* = .010*	*P* = .045*	*P* = .096
≤40	5 (71)	5 (83)	2 (29)	4 (67)
41-60	25 (74)	18 (53)	24 (77)	17 (57)
≥61	40 (61)	22 (34)	37 (66)	44 (76)
Previous experience of acupuncture, n (%)	*P* = .291	*P* = .835	*P* = .049*	*P* = .835
Acupuncture experienced, n (%)	26 (72)	16 (44)	26 (81)	21 (68)
Acupuncture-naïve, n (%)	44 (61)	27 (42)	38 (61)	42 (71)
Baseline anxious mood,^a^ n (%)	*P* = .846	*P* = 629	*P* = .055	*P* = .007*
No anxiety	30 (68)	24 (48)	25 (60)	27 (57)
Yes, little, moderate or much	36 (68)	20 (44)	39 (78)	35 (81)
Baseline depressed mood,^a^ n (%)	*P* = .986	*P* = .542	*P* = .292	*P* = .018*
No depressed mood	10 (59)	14 (48)	10 (56)	17 (61)
Yes, little, moderate or much	56 (70)	31 (46)	54 (73)	46 (73)
Baseline quality of life^a^	*P* = .545	*P* = .406	*P* = .543	*P* = .334
High to moderate	54 (70)	40 (49)	51 (70)	51 (68)
Low to very low; none-existing	12 (60)	5 (31)	13 (68)	12 (75)
Baseline optimism^a^	*P* = .047*	*P* = .679	*P* = .585	*P* = .212
I don’t try to be positive (0-2)	10 (83)	9 (50)	8 (67)	14 (82)
I try to be positive (3-4)	52 (67)	31 (42)	52 (72)	44 (65)
*Treatment related factors*				
Experienced needle-induced pain, n (%)	*P* = .107	*P* = .014*	*P* = .031*	*P* = .538
No pain	25 (60)	29 (39)	30 (79)	47 (68)
Mild pain	29 (81)	13 (68)	22 (67)	14 (74)
Moderate to much pain	10 (75)	1 (100)	7 (47)	1 (100)
Blinding statement	*P* = .428	*P* = .339	*P* = .827	*P* = .033*
Penetrating the skin	48 (66)	34 (50)	46 (68)	50 (77)
Placed against the skin	5 (83)	4 (36)	4 (80)	6 (60)
Do not know, guesses	12 (80)	5 (31)	10 (71)	6 (43)
Treating therapist,^b^ n (%)	*P* = .008*	*P* = .655	*P* = .277	*P* = .302
Therapist A, n (%)	22 (46)	16 (34)	27 (57)	4 (66)
Therapist B, n (%)	10 (91)	3 (50)	5 (45)	28 (58)

Numbers (n) and proportions (%) of patients in different subgroups reporting at least one side-effect during the treatment period are presented, with *P*-values comparing the different subgroups.

a For ordinal or continues variables, *P*-values are based on the entire scale, although categorized data are presented, for pedagogical reasons.

b Of the 215 treated patients, 113 (53%) were treated by one single therapist (95 were treated by therapist A and 18 by therapist B), 97 (45%) were treated by 2 therapists, and 5 by 3 therapists (2%).

* Statistically significant difference between the compared groups.

### Factors Modifying Nocebo Responses During Sham Acupuncture

Women (*P* = .049), younger patients (*P* = .010) and patients experiencing more needle-induced pain (*P* = .014) were more likely to experience negative side-effects of sham acupuncture than other patients (Table 3).

### Factors Modifying Positive or Negative Side-Effects of Genuine Acupuncture

Patients older than 40 years (*P* = .045), patients who experienced less needle-induced pain (*P* = .008), and patients with previous experiences of acupuncture (*P* = .049) were more likely to report positive side-effects of genuine acupuncture. More patients with a less optimistic attitude reported negative side-effects than patients with an optimistic attitude (*P* = .047). Higher proportions of patients treated with genuine acupuncture by therapist B reported negative side-effects compared to patients treated by therapist A (*P* = .008; Table 3).

### The Relation Between Placebo and Nocebo Responses and Quality of Life

Sham-treated patients who experienced positive side-effects during the treatment period experienced better quality of life at the end of the period (m ± SD 64 ± 26.1 mm VAS) than patients who did not experience positive side-effects according to a bivariate analysis (m 48 ± 25.5; 16 mm difference, *P* = .020 Student’s *t*-test), 95% Confidence Interval 3.2-28.8 mm difference. In the linear regression model, the difference was still statistically significant (*P* = .048). In the genuine acupuncture group, quality of life did not differ between patients experiencing positive side-effects and patients who did not, nor differed quality of life in patients experiencing or not experiencing negative side-effects (Table 4).

**Table 4.** Quality of Life at the End of the Treatment Period in Patients Who Experienced Positive or Negative Side-Effects During Genuine or Sham Acupuncture.

	Genuine acupuncture, n = 70^a^		Sham acupuncture, n = 81^a^		Genuine acupuncture, n = 70^a^		Sham acupuncture, n = 81^a^	
Variable	Yes, negative side effects occurred n = 49	No, negative side effects did not occur n = 21	Yes, negative side effects occurred n = 36	No, negative side effects did not occur n = 45	Yes, positive side effects occurred n = 52	No, positive side effects did not occur n = 18	Yes, positive side effects occurred n = 57	No, positive side effects did not occur n = 49
QoL, mm at VAS	*P* = .728		*P* = .197		*P* = .620		*P* = .020*	
Md, 25th-75th percentile	55, 31-77	54, 37-77	63, 35-75	46, 32-70	63, 33-81	55, 32-75	63, 43-87	49, 27-69
M ± SD	55 + 27.4	59 + 24	57 + 25.8	49 + 25.7	59 + 25.4	55 + 27.2	64 + 26.1	48 + 25.5
Range	0-100	25-100	8-100	14-94	14-94	12-100	0-100	0-100

Level of QoL, Quality of Life, at the end of the treatment period in Md = median with 25th to 75th percentile and M = mean with SD = Standard Deviation at VAS = Visual Analog Scale 0 (worst imaginable) – 100 (best imaginable QoL) in mm, millimeters.

a n = 70 in the genuine acupuncture group and n = 81 in the sham acupuncture rated QoL.

* Statistically significant difference between the compared groups, according to a bivariate test. According to a linear regression model, *P* = .048.

## Discussion

This study found that placebo and nocebo effects commonly occurred during acupuncture therapy since neither positive nor negative side-effects differed between patients receiving genuine or sham acupuncture. Factors increasing the *placebo response* during sham acupuncture were positive treatment expectation, female sex, anxious mood and depressed mood, successful blinding, and a data collection method exemplifying positive side-effects. Factors increasing the *nocebo response* during sham acupuncture were female sex, younger age, and experiences of needle-induced pain during treatment. The patients who experienced placebo-effects in terms of positive side-effects of sham acupuncture experienced better quality of life compared to patients who did not experience positive side-effects.

The observation that placebo and nocebo effects commonly occurred during acupuncture treatments seems interesting in the light of previous studies of side effects during acupuncture that were not sham-controlled, presenting the positive side effects as if they were a result of the acupuncture itself.^19,38^ The lack of differences in the variety of positive and negative side-effects in the given study context may lead researchers and clinicians to wonder if the levels of positive and negative side-effects reported in previous studies were related to specific mechanisms of acupuncture or partly were placebo and nocebo responses. Of 6348 patients, a quarter reported tiredness and 3 quarters reported relaxation after genuine acupuncture treatments in general.^19^ In the present study, 42% of both genuine and sham acupuncture treated patients experienced tiredness and relaxation was experienced by 59% in the genuine and 57% in the sham acupuncture group. The present study revealed that neither the positive nor the negative side-effects were results of the genuine acupuncture mechanisms such as skin-penetration and needle-stimulation on a traditional acupuncture point in the current setting. The study patients reported for example hematoma and soreness after sham acupuncture, although the sham needles did not induce bleeding or nociceptive input explaining the soreness.^29,30^ However, nocebo manipulation may modulate short-term pain perception and activate operculum over an extended period.^39^ The observation that placebo and nocebo effects commonly occurred during sham acupuncture was in line with a previous study, presenting a dramatic effect of sham acupuncture, especially when given by therapists adopting an empathic interaction with the patients.^20^ The present study suggests that non-specific components of the acupuncture therapy have greater impact on level of side-effects than the specific mechanisms of acupuncture treatment. This highlights the important role of the context surrounding a treatment not only for inducing improvements in the *intended* treatment outcome but also for minimizing negative side-effects and maximizing positive side-effects.^3^ Research have highlighted that the patient-clinician relationship seems to be a key component for successful treatment, across several medical disciplines and treatment modalities.^4,5,7,40^ Acupuncture is considered a therapist intensive treatment modality, with an extensive interpersonal contact surface, and has been criticized for obscuring any “genuine” treatment effects.^20,21^ However, the patient-therapist interaction also contributed to greater variance of the treatment outcomes than the difference between a variety of drugs and placebo during pharmacological therapies in depressed patients.^41^ Accordingly, nonspecific effects may play an important role in integrative cancer care across treatment modalities. In line with the present study observation regarding nocebo responses, patients who receive placebo pills,^41,42^ placebo vaccines,^43^ or placebo manual therapies^40^ also often report a high frequency of adverse events, that is, nocebo responses.

Factors associated with *placebo response* during sham acupuncture – positive treatment expectation, female sex, anxious mood and depressed mood, successful blinding, and a data collection method exemplifying positive side effects – may be discussed in relation to findings seen in previous studies. The patients’ treatment-expectations regarding effects on the intended outcome, emesis, also highly modified also the placebo response. This is in line with the general theories on mechanisms for inducing placebo responses,^3,6^ and in line with other acupuncture studies showing that expectations highly modify intended treatment outcomes.^17,18,21,44^ However, there are also observations on a lack of relation between treatment expectations and placebo responses, for example, in an experimental study of pain,^16^ and in some efficacy studies of acupuncture.^45-47^ An intervention study strengthened half of the patients to consider that their antiemetic acupressure therapy was effective, irrespective of if it was a genuine or a sham acupressure therapy. The patients who before the therapy highly expected themselves to become nauseous needed to consume less rescue antiemetics after the expectancy-strengthening information instead of just neutral information. However, patients who did not expect themselves to become nauseous experienced more nausea if they had strengthened information instead of just neutral information, possibly because the information provided made them devote extra attention to the risk for nausea.^48^ The observation that dramatically higher proportions of patients reported positive side-effects in the genuine and the sham acupuncture group when using examples of positive side-effects than when reported using the patients’ own worlds, highlights the effect of inducing expectations by the data collection per se.^49^ Further, the observation that blinding success was relevant for the placebo response is probably related to the blinded patients’ expectations to receive an effective, genuine, treatment. A previous study indicated that the risk for receiving sham acupuncture in sham-controlled trials may affect the effect of genuine acupuncture, compared to when giving acupuncture in routine care settings.^50^ The finding that factors other than expectancy-related factors also increased placebo responses bring new knowledge. Over time, since the first rather controversial study highlighted characteristics of individuals more likely than others to respond to placebo pills,^51^ studies have suggested a variety of characteristics associated with placebo responses without reaching any consensus.^52-55^ In line with the current study, previous studies presented that female sex increased the placebo response in people with chronic pain^56^ and during sham acupuncture given by a therapist adopting an “empathic” therapist style,^57^ respectively. In the present study, pre-treatment anxious mood increased the placebo response, which was in line with the very first study regarding characteristics of individuals responding to placebo.^51^ High level of anxiety was associated with greater placebo response regarding relief of radiating leg pain in patients with low back pain.^54^ However, other studies did not observe a such association,^57^ a recently published systematic review included.^58^ Optimism was associated with higher placebo response regarding pain relief in another study.^59^ The present study did not reveal such a relationship regarding placebo response of sham acupuncture. However, optimism was related to higher occurrence of positive side-effects of genuine acupuncture. In patients receiving genuine acupuncture, previous experience of acupuncture was related to higher occurrence of positive side-effects than in acupuncture-naïve patients. Learning from previous situations and thus forming expectations is a key component in general theories on mechanisms for shaping new experiences,^8,16^ and the present study confirms this key component in this clinical context.

Factors increasing the *nocebo response* during sham acupuncture were female sex, younger age, and experiences of needle-induced pain during treatment, while anxiety was not associated with the nocebo response. There seem to be no consensus for either sex or anxiety as factors modifying nocebo responses.^10,52,53^ In line with the current study, younger age increased the sensitivity for developing the negative side-effect nausea during chemotherapy^60^ and increased the nocebo response to placebo pills in the psychiatric setting.^61^ The observation that higher levels of needle-induced pain were related to higher occurrence of nocebo responses of sham acupuncture and to lower occurrence of positive side-effects of genuine acupuncture was in line with previous reviews, promoting the impact of a pleasant context of the treatment.^54^ Regarding neurofunctional pain anticipation mechanisms, brain imaging studies show neural activations of cortical systems associated with pain experience, even without nociceptive stimulation. Potentially dangerous stimuli, such as a painful stimulus, elicit unpleasant stress responses important for avoiding tissue damage.^62^ Hypothetically, that kind of mechanism may be a part of the explanation of the role of needle-induced pain for inducing unpleasant nocebo responses during sham acupuncture. Further, the patients might have thought that the more pain the needling induced, the more “powerful” would it be to induce negative side effects. In line with this, a more “powerful” placebo treatment in terms of colorful placebo pills induced more responses than less “powerful” ordinary white placebo pills.^63^ The observation that the therapist was relevant for modifying the patients’ responses was in line with previous study findings regarding the *intended* treatment outcomes; the treatment outcomes differed markedly between the therapists, despite standardization.^57^ The present study pays attention to the role of the therapist also for modifying the level of adverse effects during sham acupuncture in terms of nocebo responses.

The patients who experienced placebo responses in terms of positive side effects of sham acupuncture experienced better quality of life compared to patients who did not experience positive side-effects. The difference remained after controlling for other factors that plausibly contribute to variations in quality of life. However, other confounding factors, that researchers were not aware of and that were thus not measured or controlled for may also have contributed to the difference. The better quality of life in the sham-treated patients experiencing placebo responses indicated the clinical relevance of the placebo responses.^22^ Placebo responses may be associated by neurobiological responses, for example endogenous regulation of endocrine and immune systems, resulting in health promoting effects.^64^ Health-care practitioners may be seen as agents that can induce health effects through mechanisms associated with placebo responses^65^ in integrative cancer care.^1,2^

There are several methodological issues to discuss. The data on positive and negative side-effects were derived from a RCT without any untreated control group. Three-armed acupuncture studies randomizing patients to acupuncture, sham acupuncture, or a control group receiving no acupuncture, risk less positive treatment expectancy in the control group, since the patients may be disappointed on the lack of acupuncture treatment. If the current study would have considered the placebo response to be the difference between the positive side-effects occurring in the sham group with those occurring in the untreated group, the latter group’s disappointment, that is, negative expectations, may then plausibly affect the results, causing the study to overestimate the placebo responses of the sham acupuncture treatment. A strength is thus the RCT design, allowing for comparisons not biased by group-based differences in baseline expectancy. Other strengths are the blinding success resulting from the use of a credible sham device, and the high patient compliance, resulting in a rather large number of treatment observations; 2426 treatments given to 215 patients. The study randomly allocated the patients to genuine or sham acupuncture, which succeeded in avoiding an imbalance between the groups of patient characteristics important for the studied outcomes. The lack of differences between the genuine and the sham groups regarding the variety of side effects did not depend on lack of statistical power; the study encountered preformed criteria for statistical power in the original study^26^ and the analyses detected statistically significant differences in occurrence of side-effects between different subgroups of patients. The data analysis statistically treated the VAS as a continuous scale as most other researchers do,^37^ although VAS is an ordinal scale. The interpretation of the VAS analysis did not change using Mann Whitney U-test (just for the discussion, p-values not shown in the result section) instead of Student’s t-test, indicating that the observation was robust. The original study presented that the patients were successfully blinded^26^ using the credible^24^ Park’s sham device.^29^ The observation that more of the patients who believed they received penetrating acupuncture reported positive side-effects than others, irrespective if they received genuine acupuncture or not, strengthens the importance of using a credible control. The acupuncture-delivering therapists were not blinded to acupuncture type since the therapists placed the sham needles to other points than the genuine acupuncture needles and noticed when the specific needle sensation during manipulation of genuine acupuncture needles was reached. A weakness of this paper is the lack of assessment of therapist characteristics, such as personality traits or trait empathy, which may have contributed to a better understanding of the role of the therapist^7,57,66^ for modifying treatment responses. With increasing evidence for emotional contagion based on the role of mirror neurons and other aspects of empathy^67,68^ or role of pleasant touch during acupuncture,^69^ further studies are welcomed, looking at the therapist’s role in conveying intended and non-intended effects of treatments.

The placebo and nocebo response exists in a wide range of therapies,^4,5,7,16,36,40,55,56,58,68^ not only in integrative cancer therapies. By the insights from placebo and nocebo mechanism studies, it may be possible to further understand variability in the placebo and nocebo components of the overall benefit associated with a treatment. These new insights may be used to improve research methodology applied in efficacy studies and to optimize the efficacy of treatments in clinical practice.^6,11,68^ This paper increases the knowledge regarding placebo and nocebo responses in an integrative cancer therapy context. Some of the factors that were found to maximize placebo responses and minimize nocebo responses in the current study were factors that the therapist plausibly may affect, that is, positive treatment expectation and lack of needle-induced pain. This implies that acupuncture-delivering therapists striving to maximize placebo-responses and minimize nocebo-responses may consider strengthening the patient’s treatment expectations, and offer a pleasant, pain-free, acupuncture treatment.

## References

[1] Mao JJ Pillai GG Andrade CJ , et al. Integrative oncology: Addressing the global challenges of cancer prevention and treatment. CA Cancer J Clin. 2022;72:144-164. doi:10.3322/caac.21706 34751943 PMC13183357

[2] Witt CM Balneaves LG Cardoso MJ , et al. A comprehensive definition for integrative oncology. Natl Cancer Inst Monogr. 2017;2017:lgx012. doi:10.1093/jncimonographs/lgx012 29140493

[3] Evers AWM Colloca L Blease C , et al Consortium of Placebo Experts. What should clinicians tell patients about placebo and nocebo effects? Practical considerations based on expert consensus. Psychother Psychosom. 2021;90:49-56. doi:10.1159/000510738 33075796

[bibr4-15347354241300068] Kelley JM Kraft-Todd G Schapira L Kossowsky J Riess H. The influence of the patient-clinician relationship on healthcare outcomes: a systematic review and meta-analysis of randomized controlled trials. PLoS One. 2014;9:e94207. doi:10.1371/journal.pone.0094207 PMC3981763 24718585

[bibr5-15347354241300068] Howick J Moscrop A Mebius A , et al. Effects of empathic and positive communication in healthcare consultations: a systematic review and meta-analysis. J R Soc Med. 2018;111:240-252. 29672201 10.1177/0141076818769477 PMC6047264

[6] Caliskan EB Bingel U Kunkel A. Translating knowledge on placebo and nocebo effects into clinical practice. Pain Rep. 2024;9:e1142. doi:10.1097/PR9.0000000000001142 PMC10965200 38533458

[bibr7-15347354241300068] Blasini M Peiris N Wright T Colloca L. The role of patient-practitioner relationships in placebo and Nocebo phenomena. Int Rev Neurobiol. 2018;139:211-231. doi:10.1016/bs.irn.2018.07.033 30146048 PMC6176716

[bibr8-15347354241300068] Colloca L. The Nocebo effect. Annu Rev Pharmacol Toxicol. 2024;64:171-190. doi:10.1146/annurev-pharmtox-022723-112425 37585661 PMC10868531

[bibr9-15347354241300068] Isawa M Kajiyama M Tominaga Y , et al. Review of clinical studies on the nocebo effect. Pharmazie. 2020;75:548-553. doi:10.1691/ph.2020.0642 33239127

[10] Rooney T Sharpe L Todd J Richmond B Colagiuri B. The relationship between expectancy, anxiety, and the nocebo effect: a systematic review and meta-analysis with recommendations for future research. Health Psychol Rev. 2023;17:550-577. doi:10.1080/17437199.2022.2125894 36111435

[11] Vase L. Can insights from placebo and nocebo mechanisms studies improve the randomized controlled trial? Scand J Pain. 2020;20:451-467. doi:10.1515/sjpain-2019-0183 32609651

[12] Lee SM Choi HC Hyun MK. An overview of systematic reviews: complementary therapies for cancer patients. Integr Cancer Ther. 2019;18:1534735419890029. doi:10.1177/1534735419890029 31876212 PMC6933541

[13] Guo J Pei L Chen L , et al. Research Trends of acupuncture therapy on cancer over the past two decades: a bibliometric analysis. Integr Cancer Ther. 2020;19:1534735420959442. doi:10.1177/1534735420959442 33143477 PMC7675915

[14] De Melo MN Pai P Lam MOY , et al. The provision of complementary, alternative, and integrative medicine information and services: a review of world leading oncology hospital websites. Curr Oncol Rep. 2022;24:1363-1372. doi:10.1007/s11912-022-01296-y 35639330

[15] Huemer M Graca S Bitsche S , et al. Mapping the clinical practice of traditional, complementary and integrative medicine in oncology in Western countries: a multinational cross-sectional survey. J Integr Med. 2024;22:64-71. doi:10.1016/j.joim.2023.12.002 38199884

[16] Colloca L Akintola T Haycock NR , et al. Prior therapeutic experiences, not expectation ratings, predict placebo effects: an experimental study in chronic pain and healthy participants. Psychother Psychosom. 2020;89:371-378. doi:10.1159/000507400 32492688 PMC7581546

[17] Colagiuri B Smith CA. A systematic review of the effect of expectancy on treatment responses to acupuncture. Evid Based Complement Alternat Med. 2012;2012:857804. doi:10.1155/2012/857804 22203882 PMC3235945

[18] Hayden JA Wilson MN Riley RD , et al. Individual recovery expectations and prognosis of outcomes in non-specific low back pain: prognostic factor review. Cochrane Database Syst Rev. 2019;2019:CD011284. doi:10.1002/14651858.CD011284.pub2 PMC6877336 31765487

[19] Odsberg A Schill U Haker E. Acupuncture treatment: side effects and complications reported by Swedish physiotherapists. Complement Ther Med. 2001;9:17-20. doi:10.1054/ctim.2000.0418 11264965

[20] Kaptchuk TJ Kelley JM Conboy LA , et al. Components of placebo effect: randomised controlled trial in patients with irritable bowel syndrome. BMJ. 2008;336:999-1003. doi:10.1136/bmj.39524.439618.25 18390493 PMC2364862

[bibr21-15347354241300068] Enblom A Lekander M Hammar M , et al. Getting the grip on nonspecific treatment effects: Emesis in patients randomized to acupuncture or sham compared to patients receiving standard care. PLoS One. 2011;6:e14766. 10.1371/journal.pone.0014766 PMC3063156 21448267

[bibr22-15347354241300068] Koog YH Lee JS Wi H. Clinically meaningful nocebo effect occurs in acupuncture treatment: a systematic review. J Clin Epidemiol. 2014;67:858-869. doi:10.1016/j.jclinepi.2014.02.021 24780405

[23] Porporatti AL Costa YM Réus JC , et al. Placebo and nocebo response magnitude on temporomandibular disorder-related pain: a systematic review and meta-analysis. J Oral Rehabil. 2019;46:862-882. doi:10.1111/joor.12827 31155735

[24] Enblom A Johnsson A Hammar M Steineck G Börjeson S. The nonpenetrating telescopic sham needle may blind patients with different characteristics and experiences when treated by several therapists. Evid Based Complement Alternat Med. 2011;2011:185034. doi:10.1155/2011/185034 21747890 PMC3124016

[25] Mao JJ Bowman MA Xie SX , et al. Electroacupuncture versus gabapentin for hot flashes among breast cancer survivors: a randomized placebo-controlled trial. J Clin Oncol. 2015;33:3615-3620. doi:10.1200/JCO.2015.60.9412 26304905 PMC4622101

[26] Enblom A Johnsson A Hammar M , et al. Acupuncture compared with placebo acupuncture in radiotherapy-induced nausea–a randomized controlled study. Ann Oncol. 2012;23:1353-1361. doi:10.1093/annonc/mdr402 21948812

[27] Widgren Y Silén M Wåhlin I , et al. Chemotherapy-induced emesis: experienced burden in life, and significance of treatment expectations and communication in chemotherapy care. Integr Cancer Ther. 2023;22:15347354231217296. doi:10.1177/15347354231217296 PMC10725131 38098295

[28] Lagerstedt K Efverman A. A randomized sham-controlled mixed methods pilot study of the feasibility of acupuncture for chemotherapy-induced neuropathy: lessons learned from patient experiences in integrative cancer care. Integr Cancer Ther. 2023;22:15347354231178877. doi:10.1177/15347354231178877 37294052 PMC10262658

[29] Park JJ. Developing and validating a sham acupuncture needle. Acupunct Med. 2009;27:93. doi:10.1136/aim.2009.001495 19734377

[30] Scheffold BE Hsieh CL Litscher G. Neuroimaging and neuromonitoring effects of electro and manual acupuncture on the central nervous system: a literature review and analysis. Evid Based Complement Alternat Med. 2015;2015:641742. doi:10.1155/2015/641742 26339269 PMC4538975

[31] Skoogh J Ylitalo N Larsson Omeróv P , et al.; Swedish-Norwegian Testicular Cancer Group. ‘A no means no’–measuring depression using a single-item question versus hospital anxiety and depression scale (HADS-D). Ann Oncol. 2010;21:1905-1909. doi:10.1093/annonc/mdq058 20231301

[32] Bowling A. Just one question: if one question works, why ask several? J Epidemiol Community Health. 2005;59:342-345. doi:10.1136/jech.2004.021204 15831678 PMC1733095

[33] Watson M Greer S Young J , et al. Development of a questionnaire measure of adjustment to cancer: the MAC scale. Psychol Med. 1988;18:203-209. doi:10.1017/s0033291700002026 3363039

[34] Efverman A. A single-item expectancy measure’s validity, reliability, and responsiveness to detect changes in clinical efficacy studies of integrative cancer therapies: a methodology study. Integr Cancer Ther. 2024;23:15347354241273944. doi:10.1177/15347354241273944 39164885 PMC11339744

[35] Enblom A Johnsson A. Type and frequency of side effects during PC6 acupuncture: observations from therapists and patients participating in clinical efficacy trials of acupuncture. Acupunct Med. 2017;35:421-429. doi:10.1136/acupmed-2016-011270 29222203

[36] Wang S Xiong Z Cui Y , et al. Placebo and nocebo responses in pharmacological trials of tic disorders: a meta-analysis. Mov Disord. 2024;39:585-595. doi:10.1002/mds.29714 38247265

[37] Williamson A Hoggart B. Pain: a review of three commonly used pain rating scales. J Clin Nurs. 2005;14:798-804. doi:10.1111/j.1365-2702.2005.01121.x 16000093

[38] Wu J Hu Y Zhu Y , et al. Systematic review of adverse effects: a further step towards modernization of acupuncture in China. Evid Based Complement Alternat Med. 2015;2015:1-19. doi:10.1155/2015/432467 PMC4538973 26339265

[39] Ellerbrock I Wiehler A Arndt M May A. Nocebo context modulates long-term habituation to heat pain and influences functional connectivity of the operculum. Pain. 2015;156:2222-2233. doi:10.1097/j.pain.0000000000000297 26181304

[40] Ezzatvar Y Dueñas L Balasch-Bernat M Lluch-Girbés E Rossettini G. Which portion of physiotherapy treatments' effect is not attributable to the specific effects in people with musculoskeletal pain? A meta-analysis of randomized placebo-controlled trials. J Orthop Sports Phys Ther. 2024;54:391-399. doi:10.2519/jospt.2024.12126 38602164

[41] Furukawa TA Cipriani A Atkinson LZ , et al. Placebo response rates in antidepressant trials: a systematic review of published and unpublished double-blind randomised controlled studies. Lancet Psychiatry. 2016;3:1059-1066. doi:10.1016/S2215-0366(16)30307-8 27726982

[42] Zhang Y Xu Y Liu S , et al. The nocebo response in pharmacologic treatments of primary headache: A systematic review and meta-analysis. J Clin Pharmacol. 2022;62:1257-1272. doi:10.1002/jcph.2072 35532312

[43] Haas JW Bender FL Ballou S , et al. Frequency of adverse events in the placebo arms of COVID-19 vaccine trials: a systematic review and meta-analysis. JAMA Netw Open. 2022;5:e2143955. doi:10.1001/jamanetworkopen.2021.43955 PMC8767431 35040967

[44] Efverman A. Treatment expectations seem to affect bowel health when using acupuncture during radiotherapy for cancer: secondary outcomes from a clinical randomized sham-controlled trial. Complement Ther Med. 2020;52:102404. doi:10.1016/j.ctim.2020.102404 32951698

[45] Ee CC Thuraisingam S Pirotta MV , et al. Expectancy after the first treatment and response to acupuncture for menopausal hot flashes. PLoS One. 2017;12:e0186966. doi:10.1371/journal.pone.0186966 PMC5659680 29077767

[bibr46-15347354241300068] Barth J Muff S Kern A , et al. Effect of briefing on acupuncture treatment outcome expectations, pain, and adverse side effects among patients with chronic low back pain: A randomized clinical trial. JAMA Netw Open. 2021;4:e2121418. doi:10.1001/jamanetworkopen.2021.21418 PMC8433606 34505889

[47] Li X Baser RE Bryl K , et al. How does pretreatment expectancy influence pain outcomes with electroacupuncture and battlefield acupuncture in cancer survivors?: Pretreatment expectancy and pain reduction by acupuncture. Integr Med Res. 2024;13:101040. doi:10.1016/j.imr.2024.101040 PMC11077026 38721341

[48] Roscoe JA O'Neill M Jean-Pierre P , et al. An exploratory study on the effects of an expectancy manipulation on chemotherapy-related nausea. J Pain Symptom Manag. 2010;40:379-390. 10.1016/j.jpainsymman.2009.12.024 PMC3156553 20579837

[49] Hansen E Zech N. Nocebo effects and negative suggestions in daily clinical practice - forms, impact and approaches to avoid them. Front Pharmacol. 2019;10:77. doi:10.3389/fphar.2019.00077 30814949 PMC6381056

[50] Kim TH Lee MS Alraek T Birch S. Acupuncture in sham device controlled trials may not be as effective as acupuncture in the real world: a preliminary network meta-analysis of studies of acupuncture for hot flashes in menopausal women. Acupunct Med. 2020;38:37-44. doi:10.1136/acupmed-2018-011671 31517500 PMC7041625

[51] Lasagna L Mosteller F Von Felsinger JM Beecher HK. A study of the placebo response. Am J Med. 1954;16:770-779. doi:10.1016/0002-9343(54)90441-6 13158365

[52] Lou JS. Placebo responses in Parkinson's disease. Int Rev Neurobiol. 2020;153:187-211. doi:10.1016/bs.irn.2020.03.031 32563288

[bibr53-15347354241300068] Bizzi F Voltolini S Fiaschi MD Cavanna D. Assessing clinical and psychological features: who are patients showing a nocebo re-action during the drug challenge test? Eur Ann Allergy Clin Immunol. 2019;51:258-265. doi:10.23822/EurAnnACI.1764-1489.116 31594299

[bibr54-15347354241300068] Wasan AD Kaptchuk TJ Davar G Jamison RN. The association between psychopathology and placebo analgesia in patients with discogenic low back pain. Pain Med. 2006;7:217-228. doi:10.1111/j.1526-4637.2006.00154.x 16712621

[55] Wang Y Chan E Dorsey SG Campbell CM Colloca L. Who are the placebo responders? A cross-sectional cohort study for psychological determinants. Pain. 2022;163:1078-1090. doi:10.1097/j.pain.0000000000002478 34740998 PMC8907332

[56] Olson EM Akintola T Phillips J , et al. Effects of sex on placebo effects in chronic pain participants: a cross-sectional study. Pain. 2021;162:531-542. doi:10.1097/j.pain.0000000000002038 32826757 PMC7854995

[57] Kelley JM Lembo AJ Ablon JS , et al. Patient and practitioner influences on the placebo effect in irritable bowel syndrome. Psychosom Med. 2009;71:789-797. doi:10.1097/PSY.0b013e3181acee12 19661195 PMC2818141

[58] Kang H Miksche MS Ellingsen DM. Association between personality traits and placebo effects: a preregistered systematic review and meta-analysis. Pain. 2023;164:494-508. doi:10.1097/j.pain.0000000000002753 35947877

[59] Geers AL Wellman JA Fowler SL Helfer SG France CR. Dispositional optimism predicts placebo analgesia. J Pain. 2010;11:1165-1171. doi:10.1016/j.jpain.2010.02.014 20627818 PMC2956003

[60] Meissner K Talsky N Olliges E , et al. Individual factors contributing to nausea in first-time chemotherapy patients: a prospective cohort study. Front Pharmacol. 2019;10:410. doi:10.3389/fphar.2019.00410 31133847 PMC6524707

[61] Dodd S Walker AJ Brnabic AJM , et al. Incidence and characteristics of the nocebo response from meta-analyses of the placebo arms of clinical trials of olanzapine for bipolar disorder. Bipolar Disord. 2019;21:142-150. doi:10.1111/bdi.12662 29926533

[62] Palermo S Benedetti F Costa T Amanzio M. Pain anticipation: an activation likelihood estimation meta-analysis of brain imaging studies. Hum Brain Mapp. 2015;36:1648-1661. doi:10.1002/hbm.22727 25529840 PMC6869158

[63] Kong J Spaeth R Cook A , et al. Are all placebo effects equal? Placebo pills, sham acupuncture, cue conditioning and their association. PLoS One. 2013;8:e67485. doi:10.1371/journal.pone.0067485 PMC3729687 23935833

[64] Wager TD Atlas LY. The neuroscience of placebo effects: connecting context, learning and health. Nat Rev Neurosci. 2015;16:403-418. doi:10.1038/nrn3976 26087681 PMC6013051

[65] Wilhelm M Hermann C Rief W , et al. Working with patients’ treatment expectations - what we can learn from homeopathy. Front Psychol. 2024;15:1398865. doi:10.3389/fpsyg.2024.1398865 38860049 PMC11163137

[66] Barth J Schafroth L Witt CM. Overlap and differences between patient and provider expectations for treatment outcomes: the case of acupuncture. J Pain. 2016;17:685-693. doi:10.1016/j.jpain.2016.01.477 26921463

[67] Riess H. Empathy in medicine–a neurobiological perspective. JAMA. 2010;304:1604-1605. doi:10.1001/jama.2010.1455 20940387

[68] Bajcar EA Bąbel P. Social learning of placebo effects in pain: a critical review of the literature and a proposed revised model. J Pain. 2024;25:104585. doi:10.1016/j.jpain.2024.104585 38825051

[69] Chae Y Olausson H. The role of touch in acupuncture treatment. Acupunct Med. 2017;35:148-152. doi:10.1136/acupmed-2016-011178 28151404

